# Does Cognition Have a Role in Plasticity of “Innate Behavior”? A Perspective From *Drosophila*

**DOI:** 10.3389/fpsyg.2018.01502

**Published:** 2018-08-31

**Authors:** E. Axel Gorostiza

**Affiliations:** Departamento de Farmacología, Facultad de Ciencias Químicas, Instituto de Farmacología Experimental de Córdoba-CONICET, Universidad Nacional de Córdoba, Córdoba, Argentina

**Keywords:** innate behavior, insects, behavioral flexibility, *Drosophila*, stimulus-response, behavioral plasticity

## Behavioral plasticity and the stimulus–response model

The term innate is commonly used to refer to behaviors inherited and not learned or derived from experience. This definition denies or ignores the inborn components of learning. An animal can only learn if it already has the components required for learning, e.g., the molecular and neuronal substrates. Moreover, all behaviors are, to some extent, susceptible to modification by experience. Hence, no behavior can be strictly learned or innate (Shettleworth, 2010), making this distinction and the terms scientifically inappropriate to some extent (Mameli and Bateson, 2006; Bateson and Mameli, 2007). However, given the absence of a better term, it is still possible to find some behaviors classified as innate behaviors in literature, and defined as “stereotypic patterns of movement inherited from birth that require no prior experience for proper execution” (Kim et al., 2015). Noticeably, the concept of stereotypy was arbitrarily included into the definition of innate behavior. This group includes behaviors as different as escape responses (movements performed by an animal to avoid a possible predator; Card, 2012), taxes (orienting movement of an organism directed in relation to a stimulus; Zupanc, 2010), and courtship. What these behaviors have in common is that they are dominated by innate components and preferences and seem to be stereotypic and automatic responses elicited by a defined stimulus (i.e., reflexes, senso Purves et al., 2004). They are considered sensory-motor routines driven by inborn responses to biologically relevant sensory cues. This is the base of the sensory-response model, wherein the brain only reacts to external stimuli and the behaviors are the responses (Dickinson, 1985). Although there is increasing evidence of an active role of the brain with the external stimuli exerting only a modulatory effect in humans (Raichle, 2010) and invertebrates (Gaudry and Kristan, 2009; Gordus et al., 2015), many innate behaviors in insects are still described using the sensory-response model. This interpretation led to some important aspects of innate behaviors being neglected or misinterpreted. Any behavioral researcher has experienced that these behavioral responses are far from constant among groups or single individuals from the same species, or even the same retested individual (Kain et al., 2012; Buchanan et al., 2015). Researchers work hard to control this behavioral variability by modifying their experiments. Some of these manipulations include only using animals in a certain internal or motivational state. For example, in olfactory appetitive learning in *Drosophila*, only starved animals are used and the length of the starvation period influences the results (Colomb et al., 2009). Similarly, when using the proboscis extension reflex assay, animals that did not respond to sucrose in a pretraining session or naïve animals that displayed spontaneous proboscis extension to water are discarded (Shiraiwa and Carlson, 2007). However, in our efforts to control the response of animals, we are probably curtailing the repertoire of actions we can observe, thus imposing the response we want to study onto our results. This increases the probability of that specific actions occurring and, importantly, lead us to forget the importance of variability for survival. An automatic and rigid response could soon be disadvantageous. What is an appropriate response to a given stimulus when the animal is hungry may be maladaptive when the animal is seeking a mating partner or escaping from a predator, and vice versa. The animal must evaluate its internal state and the external conditions before the most adaptive action is selected. The expected outcome is the driving force that shapes the final action (Heisenberg, 2014, 2015).

The plasticity of innate behaviors is commonly interpreted under the sensory-response model as the existence of a wider repertoire of hard-wired innate routines, each being triggered by a combination of external stimuli and internal drivers. At least two possible scenarios exist under this view. In the first one, every routine has its own neuronal substrate. Any given situation will be considered a new input, activating a specific network that should inhibit all the other routines and behaviors to promote the most adaptive behavioral output. This is the classical view of innate behaviors as hard-wired. In the second scenario, there are fewer neuronal substrates, and these are not dedicated each to a specific routine but rather define the principal features of the behavior. Other modulatory inputs refine the behavioral output of the neuronal network, resulting in a broad spectrum of routines for a behavior. It is possible that both scenarios coexist. The first one could be possible for very simple behaviors with relatively little variation in their inputs. The alternative scenario represents one of the possibilities of how the internal state or the analyses of the internal state vs. external factors modulate a behavior. We do not yet fully understand how internal and external stimuli are integrated, nor how the networks that integrate those factors interact with the networks that trigger behaviors. We also do not know what each type of decision looks like at the neuronal level. It is always a possibility that what we perceive at an observational level as the activation of one of two possible mutually exclusive behaviors shares a lot at the neuronal level with what we describe as a complex decision. It is in this gap in understanding that I believe we could start thinking about whether and how cognitive processes could shape the final action in some innate behaviors.

*Drosophila* is an excellent model organism for this type of study. Their small size, their great repertoire of behaviors, and the availability of advanced genetic tools (reviewed in Owald et al., 2015; Luo et al., 2018) give us the ability to address these questions at the level of behavior, circuits, and individual cells. Current technology allows us to specifically and reversibly manipulate one or several neurons in living, behaving flies. This makes it possible to dissect the circuits dedicated to behavioral flexibility, decisions, and cognitive processes, and see how different, or not, they are at the neuronal level, and how common they are for different behavioral choices.

Menzel et al. (2007) defined cognition as the “use and handling of knowledge, which allows the animal to decide between different options in reference to the expected outcome of its potential actions” and provided three essential characteristics of cognition as part of the cognitive components of behavior: (1) rich and cross-linked forms of sensory and motor processing; (2) flexibility and experience-dependent plasticity in choice performance; and (3) long-term adaptation of behavioral routines. The goals of this opinion article are to highlight the already known but underestimated complexity of innate behaviors and to explicitly associate these studies with the concept of cognition. Although none of the following examples fall strictly under the definition of cognition, certain aspects of the processes leading to the modulation of these behaviors are similar to the cognitive components of behavior listed above.

In *Drosophila*, the giant fibers originate in the brain, and project down contralaterally to motor neurons that control the musculature responsible for jump–flight behaviors (reviewed in Allen et al., 2006). A single spike in these neurons is normally sufficient to cause a fly to take-off, resembling a visually-evoked escape response. Consequently, giant fibers were considered command neurons for these behaviors and escape responses as reflexes. However, research conducted over the last 10 years indicates that these responses are more elaborated, extending beyond the Giant Fiber motor outputs (Card, 2012). *Drosophila* escape behavior contains a sequence of at least three maneuvers (freezing, body leaning or leg posture adjustment, and wing elevation) that end with a jump, but with some degree of independence between each maneuver, allowing the fly to stop the sequence if it chooses to Card and Dickinson (2008a,b) and Card (2012). Each step comprises the addition of new information, resulting in a more variable and carefully shaped final action. This means that even in the small temporal window before a predator reaches the fly, the insect must select from a wide range of evasive maneuvers. Recently, it has been shown that looming stimuli (possibly indicating the approach of an attacker) produce a bimodal distribution in *Drosophila* escape response—with either short or long take-offs—that can be biased toward short take-offs by increasing stimulus speed (von Reyn et al., 2014). giant fibers are necessary and sufficient for short maneuvers, while long maneuvers require a parallel pathway. Linear integration of angular velocity and angular size from looming stimuli takes place in giant fibers and derives in action selection (von Reyn et al., 2017). Hence, adult *Drosophila* escape responses involve more neural control elements than a single command neuron, allowing a variety of computational and decision steps to take place before the evasive behavior occurs. Another common defensive strategy is freezing, where the animal remains still, reducing its chances of being noticed. A new study showed that *Drosophila* flies adopt a freezing strategy in a state-dependent manner (Zacarias et al., 2018). For this study, the authors developed a different setup from the one used in the escape-behavior studies previously mentioned. Flies faced 20 repeated inescapable looming stimuli instead of a single escapable looming stimulus. Under this condition, flies rarely jumped in response to the stimulus, and most of them froze. Even the flies that initially jumped ended up modifying their defensive strategy during the experiment, since the probability of jumping decreased over the course of the stimulus presentations, and the proportion of flies freezing increased. The decision between fleeing and freezing was modulated by walking speed. If flies were grooming or moving slowly at the time of threat, they were more likely to adopt a freezing strategy. In this study, the authors also started to describe part of the network involved in freezing. Zacarias et al. (2018) perfectly illustrates how experimental conditions can promote different behavioral outputs, and how the state of the animal shapes the final action. Far from stereotypic and automatic reactions, defensive behaviors appear to be carefully calculated. In the presence of a threat, the animal begins a cost–benefit computation (e.g., to eat or to adopt a defensive strategy). If the threat is near and inescapable, the fly will freeze or flee depending on the action that was performing at the time. If it is an escapable threat, then a visually mediated motor planning will determine the direction of the escape. Next, if the fly decides to jump, at least two types of take-offs could be performed, a short one in which speed is favored over wing stability or a long one that produces a steady flight.

Perhaps even more interesting is how the presence of parasitoid wasps affects oviposition in adult flies, as a mechanism to protect their offspring from a possible future threat. Parasitoid wasps are not dangerous to adult *Drosophila*, but upon encountering female wasps, female flies adopt different strategies that include choosing food containing toxic levels of alcohol to lay their eggs (promoting the death of wasps' eggs and larvae; Kacsoh et al., 2013), and reducing oviposition rates (Lefevre et al., 2011). These behavioral switches rely on sight to sense wasps. Remarkably, the external conditions are assessed in terms of the danger they represent to their offspring and not to adult flies themselves. Similarly, by choosing food with elevated levels of ethanol, the probability of the fly's offspring being parasitized decreases, and at the same time if parasitization occurs, the fly larvae are more likely to survive (Milan et al., 2012). However, there is no instant benefit for the adults that chooses the substrate. Interestingly, neuropeptide F (NPF) and its receptor NPFR1 are involved in wasp-induced ethanol oviposition preference. NPF and NPFR1 are required for alcohol sensitivity (Wen et al., 2005), but they are also involved in the representation of the internal motivational states of hunger and satiety in the mushroom bodies via dopaminergic neurons that innervate the structure (Krashes et al., 2009). Given the preference-switch between normal food and ethanol-enriched food, and the known role of dopamine (DA) in value-based and goal-directed decision-making (Zhang et al., 2007; Schultz, 2010; Liu et al., 2012; Waddell, 2013), it would be worth investigating whether dopaminergic neurons are also recruited in this case.

Interestingly, flies form a nonassociative long-term memory of the exposure and will lay fewer eggs or choose alcohol-enriched food to lay their eggs for 24–48 h after wasp exposure (Kacsoh et al., 2013, 2015). Strikingly, it has been shown that flies visually exposed to wasps can transmit oviposition reduction behavior to naive flies (Kacsoh et al., 2015), an interesting case of social learning (Grüter and Leadbeater, 2014). That is to say, flies that never encounter a wasp can acquire and use the knowledge of others to modify their oviposition behavior. Kacsoh et al. (2015) showed that oviposition reduction behavior of naive flies (students) could last for 24 h after they were separated from wasp-exposed flies (teacher), but they could not teach others. They also demonstrated that learning mutants were unable to teach or be students but showed normal acute oviposition reduction during wasp exposure. Visual cues alone are sufficient for acute reduction in oviposition and memory formation in teachers, and social-learning responses. However, social learning requires teachers to have intact wings for students to learn, suggesting a role for both wings in communication through visual cues (Kacsoh et al., 2015). It is also noteworthy that all these learning processes require the mushroom bodies, structures previously demonstrated to be important for valence and memory-based action selection (Zhang et al., 2007; Aso et al., 2014) and to contain and receive inputs from dopaminergic and octopaminergic neurons (Zhang et al., 2007; Kim et al., 2013; Waddell, 2013; Wu et al., 2013). DA and octopamine (OA) are key modulators of behavior. OA has been implicated in state-dependent changes in visual processing (Longden and Krapp, 2009; Suver et al., 2012), experience-dependent modulation of aggression (Bonini, 2000; Stevenson et al., 2005; Hoyer et al., 2008), social decision-making (Certel et al., 2010), and reward (Burke et al., 2012). DA is also known for its roles in reward (Barron et al., 2010; Burke et al., 2012), motivation (Krashes et al., 2009; Zhang et al., 2016) and, as previously mentioned, value-based or goal-directed decision-making (Zhang et al., 2007; Liu et al., 2012; Waddell, 2013; Beeler et al., 2014). Both seem to be involved in mediating certain aspects of value albeit in different modalities or domains (Aso et al., 2010; Burke et al., 2012; Scheiner et al., 2014; Huetteroth et al., 2015).

Curiously, these two biogenic amines differently modulate phototaxis, in what it seems to be a goal-directed or value-based decision-making process. Phototaxis seems to be a special case of photopreference and manipulating the ability of flies to fly can reversibly shift it from approach to avoidance in walking flies (Gorostiza et al., 2016). Photopreference can be influenced by the shape, form, or degree of intactness of the wings, the ability of flies to move them, and the state of sensory organs related to flight. Hence, flies appear to constantly monitor their flying ability, even while walking as these experiments suggest, and adjust their photopreference accordingly. It is worth noting that the neuronal activity of dopaminergic and octopaminergic circuits is indispensable and inducing for the modulation of phototactic behavior, but with opposite effects, suggesting a potential role of DA and OA, and supporting the idea of a value-based decision-making process taking place. In this view, phototaxis is not a response, but an action selected only in rather particular circumstances after a central decision-making stage that negotiates external stimuli as well as internal demands. When flying ability is compromised, the value of the different consequences of moving toward light changes and the dangers become more prominent due to the difficulties to escape; hence, the flies choose to hide until the danger goes away or flying ability is restored. Immediately after emerging from the pupal case, all flies experience a flightless period during the wing expansion phase. In line with the results above, flies go through a phase of reduced phototaxis that extends beyond wing expansion until the stage when its wings render it capable of flying (Chiang, 1963). The alteration in flying ability may promote a shift in the expected outcome (Heisenberg, 2014, 2015), which would eventually drive the selection of an alternative, more adaptive action, as seen in preference suppression assays where air, light, and gravitaxis cues were paired with aversive stimuli (Seugnet et al., 2009; Baggett et al., 2018). In those cases, flies learn that cues that usually indicate an escape route will lead them to negative outcomes (an aversive taste or an aversive temperature). Noticeably, wing expansion in flies also involves a decision process. After emerging from the pupal case, flies select a suitable perch and expand their wings, but wing expansion can be delayed under adverse environmental conditions, e.g., space restriction (Cottrell, 2009; Peabody et al., 2009). Work in *Drosophila* uncovered part of the neuronal network involved in the decision to expand the wings, and showed the connection with the decision to perch, which required an assessment of the external factors (Peabody et al., 2009).

These examples serve to demonstrate how innate behaviors can in fact be the outcomes of complex modulatory processes, careful assessment of factors and decisions, and not mere stereotypic and automatic responses. Through these examples, we can see some aspects that resemble cognitive components (Menzel et al., 2007): rich sensory and motor processing (escape response), experience-dependent plasticity in choice performance (oviposition), and flexible and long-term adaptation of behavioral routines (photopreference). In light of this, I argue that the way we frame and refer to these behaviors must change. We should think about them as behaviors dominated by innate components or preferences. This simple paraphrase can change the focus of the innateness from the behavior to some component of it, moving also the preprogrammed conception with it. As mentioned by Menzel et al. (2007), these components or preferences “seem to be essentially useful in guiding the animals' behavior in their first confrontations with the external world.” Innate preferences could be extremely relevant in the absence of contradictory cues. Nonetheless, they are certainly not the only things determining the final shape of the behavior. When other factors add complexity to the situation, the innate component becomes diluted and lose strength, leaving only the behavior “without its innateness.” Under simple and controlled circumstances (a fly in a tube with a source of light at one end), the behavior looks like a stereotypic and automatic response (light is turned on, and in most cases the fly approaches the source of light). In this case, the innate component is the only relevant factor for the behavior. However, in complex situations (the flying ability of the fly is compromised), other factors become prominent and the innate component loses relevance or becomes maladaptive. The innate preference becomes another factor to be considered. I propose that in that complex situation, a cognitive process is engaged in the final tuning of the behavior (the fly avoids the light). Hence, cognition could prevent automatic maladaptive responses and also help fine-tune “innate routines,” depending on the combination of external stimuli and internal drivers. We should carefully consider the cognitive aspect of any behavior we study, no matter how seemingly dominated by innate components or stereotypic it looks.

## Author contributions

The author confirms being the sole contributor of this work and approved it for publication.

### Conflict of interest statement

The author declares that the research was conducted in the absence of any commercial or financial relationships that could be construed as a potential conflict of interest.
